# Jejunal Varices Bleeding in a Patient with Extensive Portomesenteric Thrombosis Secondary to Factor V Leiden Mutation: A Management Dilemma

**DOI:** 10.1155/2019/4526472

**Published:** 2019-02-06

**Authors:** Ahmad Ayash, Kamran Mushtaq, Mohamed Emad Abdul Qader, Khalid Mohsin Al-Ejji, Saad Rashid Al Kaabi, Shadi J. S. Khelfa

**Affiliations:** ^1^Department of Gastroenterology, Hamad Medical Corporation, Doha, Qatar; ^2^Department of Internal Medicine, Hamad Medical Corporation, Doha, Qatar

## Abstract

Ectopic varices are portosystemic collaterals that occur away from the gastroesophageal junction and account for 1-5% of all variceal bleeding. Its occurrence in the jejunum is rare. Most common cause of ectopic jejunal varices is portal hypertension especially in those patients who have undergone prior abdominal surgery. Portomesenteric thrombosis is a rare cause of ectopic jejunal varices. Ectopic varices are rare cause of obscure GI bleeding and hence should be always suspected in patients with history of portal hypertension who present with GI bleeding and have negative upper and lower GI endoscopies. Management of patients with ectopic varices is often very challenging and requires multidisciplinary approach. Therapeutic options include endoscopic therapy, interventional radiologic procedures, surgically creating shunting, or surgical resection. We present the case of a 52-year-old patient who was on anticoagulation for extensive portomesenteric thrombosis secondary to factor V Leiden heterozygous mutation and presented with melena and symptomatic anemia. Investigations showed bleeding jejunal varices as the cause of anemia. We discuss the therapeutic options and dilemma in the management of such cases.

## 1. Introduction

Ectopic varices are the name given to the portosystemic collaterals that are formed at sites other than the gastroesophageal junction [1]. The ectopic varices account for approximately 1-5% of all variceal bleeding. Its occurrence in the jejunum is exceedingly rare [2, 3]. Other recognized sites include duodenum, ileum, colon, rectum, biliary tree, peritoneum, umbilicus, ovary, right diaphragm, and sites of previous bowel surgeries [4].

Ectopic varices most commonly develop secondary to portal hypertension, but it can also develop because of other causes such as abdominal surgical procedures, congenital anomalies in venous outflow, certain familial syndromes, and abdominal vascular thrombosis, with the former being an odd cause of ectopic varices. Portomesenteric vein thrombosis (PMVT) causing small bowel varices has been reported a few times in literature as it can also cause secondary portal hypertension [5–7].

Intestinal ectopic varices commonly present with melena, hematochezia, or intraperitoneal bleeding. Triad of portal hypertension, prior history of abdominal surgery, and hematochezia without hematemesis characterizes source from intestinal varices [8]. We are reporting a case of bleeding jejunal varices secondary to extrahepatic portal hypertension in a patient with heterozygous factor V Leiden mutation which posed a difficult management dilemma.

## 2. Case Presentation

A 52-year-old man presented to the emergency department with 3-day history of fatigue, dizziness, dark stools, and mild generalized abdominal pain. There was no history of hematemesis, hematochezia, bleeding from any other site, or any similar prior episodes. There was no history of liver disease or NSAIDs. Patient was taking oral rivaroxaban 20 mg/day. His past medical history was significant for extensive portomesenteric thrombosis involving superior mesenteric, splenic, main portal, and right portal veins which was diagnosed 2 years ago. Extensive workup done for the cause revealed heterozygous mutation of factor V Leiden. Another workup showed normal protein C, protein S, and antithrombin III levels. Autoimmune workup, hepatitis B, hepatitis C, and HIV serology were all negative. The patient has no family history of any venous thromboembolism or other bleeding disorders.

On clinical examination he was hemodynamically stable and not in distress. Physical examination revealed marked pallor and normal abdominal examination. Digital rectal examination showed green stool with no evidence of melena at the time of examination.

**Figure 1:**
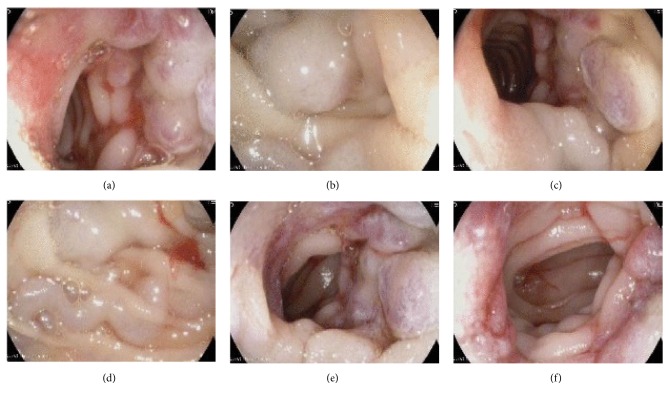
((a)-(f)) showing enteroscopy images of jejunal varices with signs of recent bleeding.

Laboratory tests revealed a hemoglobin level of 7.5 g/dl, platelet count 210,000/ul, INR 1.1, urea 6.6 mmol/l, creatinine 90 umol/l, and normal liver function tests. He was admitted as a case of probable GI bleeding. His rivaroxaban was stopped. He received transfusion of packed red blood cells for symptomatic anemia. Urgent esophagogastroduodenoscopy (EGD) was done and revealed normal esophagus and stomach; however, a suspicious area distal to 3^rd^ part of the duodenum was seen but could not be reached by the normal EGD scope. Subsequently, push enteroscopy was attempted in the same setting and revealed multiple varices in the proximal jejunum affecting a short segment with red wale signs and submucosal feeding veins (Figures 1(a)–1(f))

**Figure 2:**
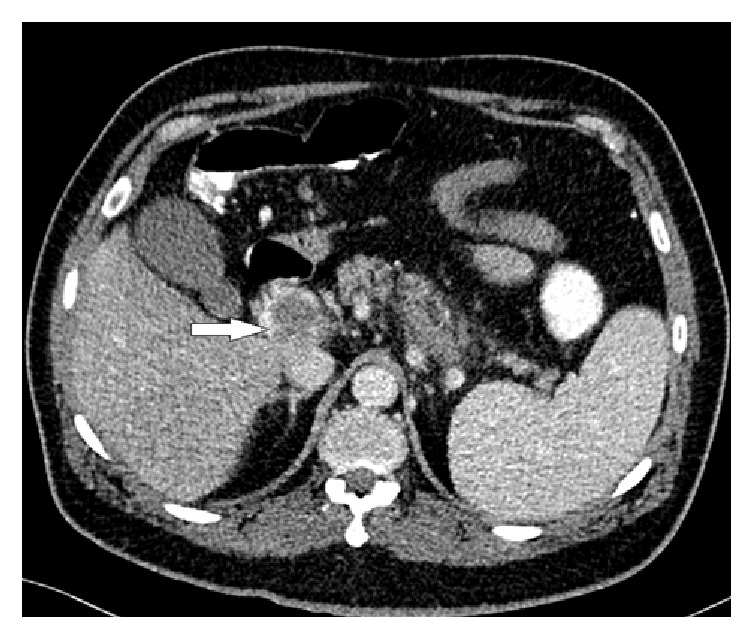
Abdomen CT scan with contrast (axial image) shows distended portal vein with filling defect, indicating thrombosis (white arrow).

**Figure 3:**
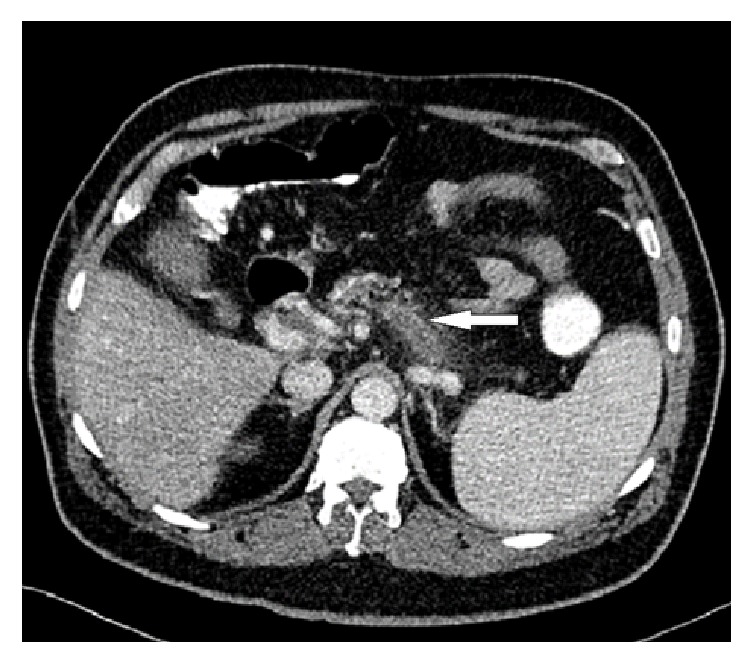
Abdomen CT scan with contrast (axial image) shows distended splenic vein with filling defect, indicating thrombosis (white arrow).

**Figure 4:**
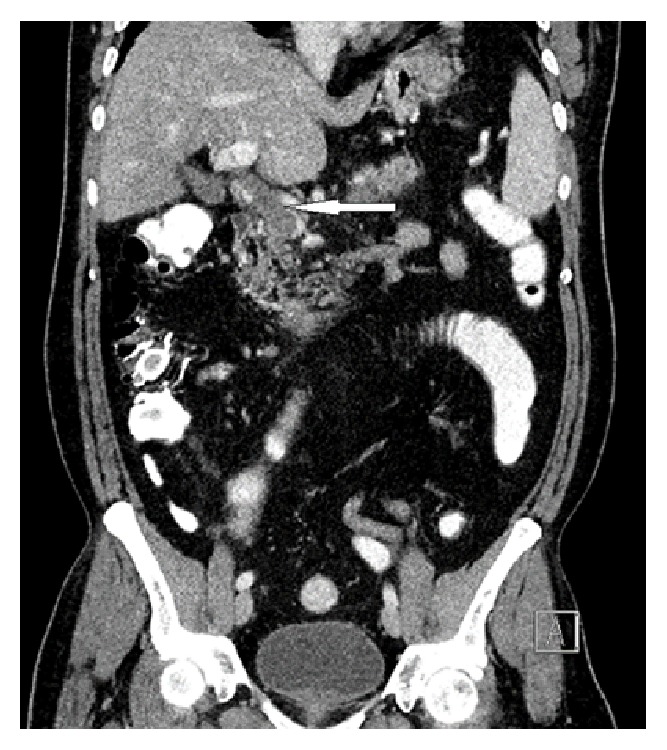
Abdomen CT scan with contrast (reformatted coronal image) shows distended portal confluence with filling defect occluding its lumen (white arrow).

CT abdomen with contrast was sought. It demonstrated total occlusion of superior mesenteric and splenic veins with well-established collateral venous circulation and reestablishment of the portal circulation at the region of porta hepatis (Figures 2, 3, and 4).

He was started on IV pantoprazole infusion and then given IV terlipressin for 3 days along with ceftriaxone 1 g/day for 5 days after the enteroscopy findings. His anticoagulation with rivaroxaban was stopped. A multidisciplinary team (MDT) meeting between general surgeon, interventional radiologist, internist, and gastroenterologist was arranged. The agreement was to surgically resect the affected small bowel segment for definitive therapy. Patient was observed in the hospital for few days and did not have any further bleeding or drop in his hemoglobin. After explaining all the benefits and risks of surgery to the patient, he decided on conservative therapy. After discussion with the hematologist, patient was kept off anticoagulation considering his high risk of GI bleeding as he did not opt for surgery. Patient was discharged home with follow-up appointment in general surgery, hematology, and gastroenterology clinics. Patient was restarted on anticoagulation with Dabigatran 110 mg twice daily by the hematologist after 4 months of bleeding event. Reduced dose was chosen due to risk of GI bleeding and the drug was chosen due to availability of the antidote idarucizumab. There was no evidence of bleeding again after resumption of the anticoagulation. Patient was doing well after eight months of follow-up, and his hemoglobin level normalized.

## 3. Discussion

Ectopic varices account for less than 5 percent of causes of variceal bleeding and jejunum is one of the least common sites. Most cases of ectopic varices are secondary to portal hypertension which is attributed to cirrhosis, whereas extra hepatic portal hypertension is very rare [5–7]. Patients with intrahepatic portal hypertension who undergo abdominal surgery are at increased risk of ectopic intestinal varices. Proposed mechanism for this is that adhesions after surgery bring the parietal surface of the abdominal organs in contact with the abdominal wall and portal hypertension results in the formation of varices below the intestinal mucosa [9].

Chronic portomesenteric vein thrombosis (PMVT) rarely can lead to occurrence of jejunal varices and carries a high mortality rate if not properly attended to [10]. Chronic portomesenteric thrombosis leads to development of venous collaterals which can bleed. This is only the second reported case of ectopic varices with mesenteric vein thrombosis attributed to heterozygous factor V Leiden mutation [6]. Factor V Leiden is one of the most common inherited thrombophilia. A heterozygous mutation puts the patient at 3-7-fold of increased risk of thrombosis, while patients with homozygous mutation have that risk increased up to 50-100-fold [11]. The management of VTE and duration of anticoagulation in patients with heterozygous factor V Leiden mutation is as in general population. However, indefinite anticoagulation is warranted in cases with life threatening VTE or at unusual sites like portal or mesenteric thrombosis. Our patient was kept on lifelong anticoagulation due to extensive PMVT.

Diagnosis of such ectopic varices is difficult and high index of suspicion is needed. Many diagnostic modalities are known to be reasonable diagnostic tools for small bowel lesions but push enteroscopy is more sensitive and carries possibilities for intervention. However, push enteroscopy does not come without risks looking at its invasive nature and the need for a trained gastroenterologist to perform it [12]. Capsule endoscopy can be used to diagnose small bowel varices but has limited role in acute GI bleeding [2].

Management of ectopic varices is the most challenging aspect of such cases. There are no clinical trials to guide the management of ectopic varices. Evidence for management is limited to a few case reports and retrospective case series. Some of the different modalities that have been used include TIPS (transjugular intrahepatic portosystemic shunts) and BRTO (balloon-occluded retrograde transvenous obliteration) [13–15]. Both carry their own risks with significant recurrence rates [9]. Other options include endoscopic band ligation and injection sclerotherapy [10]. Surgical resection of the affected bowel segment has also been reported as a treatment option in previous cases with low recurrence rates [16], but it carries its own risks especially in patients with cirrhosis (Table 1, summary of main therapeutic options in the management of ectopic varices [15]).

Wael et al. have classified the types of varices into occlusive and nonocclusive types based on hemodynamics [15]. Our patient has occlusive type of ectopic varices due to development of portal hypertension secondary to chronic portomesenteric thrombosis. The management options of occlusive type are limited to surgical or endoscopic [15]. Most data about managing ectopic varices are usually based upon patients with portal hypertension attributed to cirrhosis. In our patient interventional procedures including BRTO and TIPS were not possible looking at the extensive abdominal venous thrombosis he had, involving splenic, mesenteric, and portal veins. Glue injection was deemed high risk and difficult due to the presence of multiple large jejunal varices that will need high volume injection, so decision was to go for surgical resection of the affected small bowel segment after a multidisciplinary team meeting. Good evidence that surgery carried a very low recurrence rate was previously reported in a patient with a very similar presentation to our patient [6].

Role of beta-blockers in ectopic varices is scant. From pathophysiologic point of view, beta-blockers may seem to have role in the management of ectopic varices. However, there is no evidence to show any successful results in such cases where portal hypertension is extrahepatic in origin. The American Association for the Study of Liver Diseases Guidelines on the management of varices also recommend a multidisciplinary approach in management of bleeding ectopic varices [17].

In conclusion, isolated jejunal varices are very rare but carry a difficult diagnostic and therapeutic dilemma, so it should be managed in a multidisciplinary team with patient centered approach.

## Figures and Tables

**Table 1:** Main therapeutic options in the management of ectopic varices.

Therapeutic Options	Description	Pros	Cons
Endoscopic therapy	Use of band ligation, sclerotherapy or injection by cyanoacrylate.	Accessible. Available.	High risk of re-bleeding.

TIPS	Transjugular intrahepatic portal systemic shunting. It is a shunt creation procedure. Often combined with concomitant embolization for better success and reduction of rebleeding rates.	High success in selected patients. Can be used as primary modality or salvage therapy.	Requires Interventional radiologist. Not suitable in occlusive type of varices.

BRTO	Balloon occluded retrograde transvenous obliteration. A shunt closure procedure.	High success in gastric varices. Reported cases of success in Ectopic intestinal varices.	Requires Expert Interventional radiologist. Non-feasible in occluded type of varices.

Surgical Resection	Surgical removal of the bleeding segment	Potentially a definitive therapy. Low risk of recurrence.	Risks associated with a major intestinal surgery. Less suitable if cirrhotic. Usually a salvage therapy.
